# Nanomaterial Texture-Based Machine Learning of Ciprofloxacin Adsorption on Nanoporous Carbon

**DOI:** 10.3390/ijms252111696

**Published:** 2024-10-30

**Authors:** Maike Käärik, Nadežda Krjukova, Uko Maran, Mare Oja, Geven Piir, Jaan Leis

**Affiliations:** 1 Institute of Chemistry, University of Tartu, Ravila 14a, 50411 Tartu, Estonia; 2 Skeleton Technologies, Sepise 7, 11415 Tallinn, Estonia

**Keywords:** nanoporous carbon, antibiotics, ciprofloxacin, adsorption, machine learning, texture of nanomaterial, QnSPR

## Abstract

Drug substances in water bodies and groundwater have become a significant threat to the surrounding environment. This study focuses on the ability of the nanoporous carbon materials to remove ciprofloxacin from aqueous solutions under specific experimental conditions and on the development of the mathematical model that would allow describing the molecular interactions of the adsorption process and calculating the adsorption capacity of the material. Thus, based on the adsorption measurements of the 87 carbon materials, it was found that, depending on the porosity and pore size distribution, adsorption capacity values varied between 55 and 495 mg g^−1^. For a more detailed analysis of the effects of different carbon textures and pores characteristics, a Quantitative nano-Structure–Property Relationship (QnSPR) was developed to describe and predict the ability of a nanoporous carbon material to remove ciprofloxacin from aqueous solutions. The adsorption capacity of potential nanoporous carbon-based adsorbents for the removal of ciprofloxacin was shown to be sufficiently accurately described by a three-parameter multi-linear QnSPR equation (R^2^ = 0.70). This description was achieved only with parameters describing the texture of the carbon material such as specific surface area (*S*_dft_) and pore size fractions of 1.1–1.2 nm ($V_{N_{2}\left[1.1–1.2\right]}$) and 3.3–3.4 nm ($V_{N_{2}\left[3.3-3.4\right]}$) for pores.

## 1. Introduction

Environmental monitoring studies show that the high concentration of pharmaceutical residues in nature—both in ground and sea water—has become an actual problem. The ever-increasing environmental pollution with pharmaceutical residues is caused by the increasing human consumption of pharmaceuticals and the increasing use of veterinary drugs. The presence of antibiotics in nature creates microbial resistance because of which diseases are no longer effectively treated as harmful bacteria have adapted to the pharmaceuticals used [1,2].

**Figure 1:**
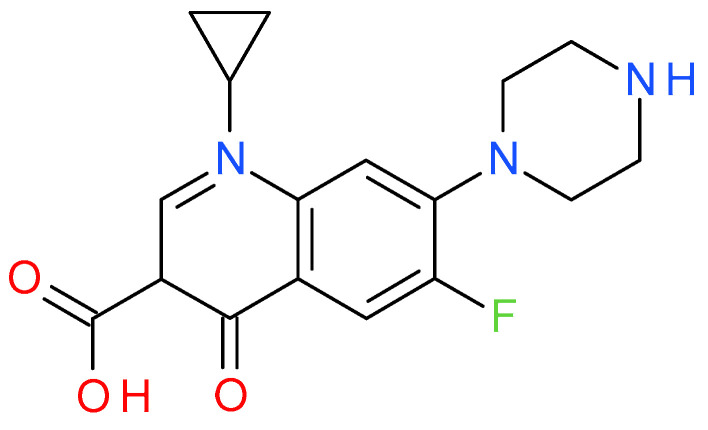
The structure of ciprofloxacin.

Ciprofloxacin (C_17_H_28_FN_3_O_3_, as shown in Figure 1) is one of the most widely used and environmentally persistent antibiotics with genotoxic properties. Ciprofloxacin belongs to the class of fluoroquinolones, which has a broad spectrum of activity against both Gram-negative and Gram-positive bacteria and is mainly used in treating humans [3,4]. It prevents the cellular reproduction of bacteria and impairs their proliferation [5]. Like other antibiotics, it can accumulate in the body, thus posing a serious health risk [6]. Ciprofloxacin is one of the drug substances found in bulk effluents that emerged from the pharmaceutical industry, and its release into the aquatic environment may present a serious threat to aquatic plants and animals [7].

The literature provides several examples of serious threats from ciprofloxacin to aquatic plants and animals. Thus, studies have shown that the growth of the microalgae *Chlamydomonas mexicana* was significantly inhibited at high ciprofloxacin concentrations (40, 60, and 100 mg L^−1^) after 11 days [8]. It has also been shown that, in the concentration range of 2–31.25 mg L^−1^, ciprofloxacin significantly inhibited the development of the green alga *Chlorella vulgaris* after a 96 h incubation period [9]. Similarly, ciprofloxacin significantly inhibited the growth of the microalgae *Pseudokirchneriella subcapitata* in the presence of an aqueous solution of quinolones [10]. In addition, ciprofloxacin has been shown to be the most toxic antibiotic for *Pseudokirchneriella subcapitata* [11]. It has also been shown that exposure to ciprofloxacin can cause metabolic disturbances, alter protein expression, and induce pathological changes in zebrafish embryos [12].

Considering ciprofloxacin’s high concentration in various aquatic environments, its stability, resistance to degradation, and potential ecotoxicity, the effective removal of ciprofloxacin before release to the environment is necessary [13]. In another study, more than 289 drug residues were found in lakes and lake sediments around the world, most of which were in Asia, Europe, and North America [14]. Of these, fluoroquinolones, including ciprofloxacin, were found at very high levels, with peak concentrations ranging from 25 µg L^−1^ to 6.5 mg L^−1^. As part of a large-scale monitoring study conducted in Europe a few years ago, 17 antibiotics were detected in treated wastewater [15]. Macrolides and fluoroquinolones were the compounds with the highest concentrations in all countries. In 2020, the results of yet another study [1] were published, confirming that drug residues, including antibiotic residues, are present in the environment of all countries that participated in the study.

Current wastewater treatment technologies can only remove treatment substance residues that are easily biodegradable or adsorbed. In contrast, the rest of the treatment substance residues pass through the treatment process almost unchanged. Therefore, the effective removal of many drug substance residues is only possible with deep cleaning technologies [16]. Many methods for removing drug substances from water have been studied: chlorination, photocatalysis, adsorption, biodegradation, oxidation, and ozonation. These methods have several drawbacks, such as high cost, high energy consumption, and the generation of toxic byproducts. The adsorption technique has several advantages over other methods: it is easy to perform, requires little energy, is effective and cost-effective, and has no toxic byproducts [17]. Therefore, the adsorption technique is a promising method for the removal of pharmaceutical residues, and it also advances the search for new (nano)-adsorbents and improves the understanding of the texture parameters of nanomaterials that cause adsorption.

Activated carbon is already used as an adsorbent today, which, together with ozonation, helps to increase the efficiency of cleaning processes in wastewater treatment and reduce expenses [16,18]. Studies have shown that the structure of the activated carbon material plays an important role in adsorption. In addition to the texture properties, the functional groups on the surface of the carbon material are also important [19]. The fact that the carbon adsorbents are quite easily regenerated is also significant. The regeneration of adsorbents either by thermal treatment [20] or different eluents [21] restores their porous properties, and the materials are effective during several cleaning cycles.

Over the last ten years, different bio-origin adsorbents have been studied to remove ciprofloxacin from aqueous solutions [22]. For example, one study found that the adsorption capacity of activated carbon made from bamboo at 25 °C is 613 mg g^−1^ [23], while the adsorption capacity of activated carbon made from peach stones is less than half of that (254 mg g^−1^) [24]. Carbon material treated with an iron oxide catalyst (Fe/C) at 30 °C showed a slight increase in adsorption capacity compared to untreated carbon, 208 mg g^−1^ and 189 mg g^−1^, respectively [25]. There are also examples where the adsorbent was prepared by pyrolysis from shrimp residues, and the adsorption capacity of 494 mg g^−1^ and 491 mg g^−1^ was achieved at pH 5 and pH 7, respectively [26]. The adsorption capacity of 885 mg g^−1^ was achieved with activated carbon obtained from the shell of pumpkin seeds [27].

Another possible carbon adsorbent is carbide-derived carbon (CDC), with a porosity and adsorption capacity that largely depend on the synthesis strategy. Some studies [28,29] showed that CDC proved to be an effective adsorbent candidate for the removal of pharmaceutical residues (including drug substances) from aqueous solutions. The principle that a higher specific surface area leads to a higher adsorption capacity is applicable to CDC.

In recent years, the use of various machine learning (ML) methods for describing the adsorption and synthesis processes of materials has grown rapidly [30]. ML, in the framework of quantitative structure–property relationships (QSPRs), creates the possibility to model the drug substances’ adsorption and the respective interactions taking place. Using different ML methods, it is possible to reveal a mathematical relationship between the parameters of the adsorption process and the structural properties of the material. Data-driven machine learning techniques can be used to classify data and predict real values. For example, ML has been used in biochar adsorption studies to speed up the search for biochar with suitable properties for drug adsorption [31,32,33,34,35,36].

The adsorption of ciprofloxacin has also been modelled using ML methods. A predictive model for ciprofloxacin adsorption has been derived for porous carbon derived from shrimp waste and commercial carbon [26]. The parameters, amount of adsorbent, pH, adsorption time and temperature, initial concentration of ciprofloxacin, NaCl concentration, and humic acid concentration, described the adsorption process and were used as descriptors to derive the ML model. The artificial neural network (ANN) was used as an ML method to establish relationships. The model correlation coefficient (R) for porous carbon from shrimp waste was 0.989, and the correlation coefficient for the commercial carbon prediction model was 0.992. The interpretation results of the models show that the initial concentrations of ciprofloxacin, pH, and the amount of adsorbent have the most significant influence on adsorption.

A more recent study compared different ML methods (XGBoost, CatBoost, LightGBM, and RF) extensively to model ciprofloxacin adsorption [33]. This study gave special attention to interpreting the models using SHapley Additive exPlanations (SHAP) analysis. The best model for predicting ciprofloxacin adsorption was obtained with the XGBoost algorithm (R^2^ = 0.89). Based on the SHAP analysis of the model descriptors, the chemical composition of the adsorbent (O/C molar ratio) has the most significant influence on the adsorption capacity, followed by the surface area and temperature at which the experiment was performed. Such findings are compatible with the results of other studies (e.g., modelling of tetracycline and sulfamethoxazole) [35].

The conclusion from the literature studies on drug substance adsorption modelling is that independent parameters for modelling are usually derived from the experimental conditions observed and recorded during the adsorption process and do not describe the structure or texture of the nanomaterial. Nanomaterial texture descriptors, such as surface area, surface groups characteristics, and average pore size or total pore volume, are rarely used in QnSPR models. This shows that the modelling of drug substance adsorption lacks a deeper insight into the porous structure, i.e., pore size distribution data, which should provide an insight into the adsorption process. On the other hand, we have previously shown that experimentally measured pore size distributions are important descriptions of adsorption behaviour in QnSPR models for double-layer capacitance of nanoporous carbon electrodes [37,38].

The present study fills the gap and focusses primarily on the influence of the adsorbent structure properties on the adsorption of ciprofloxacin, especially the texture properties, i.e., specific surface area, specific pore volume, and pore size distribution data, rather than the experimental conditions of the adsorption process. We analysed how the porosity and pore size distribution of nanoporous carbon adsorbents affect their adsorption capacity under specific test conditions. For this purpose, a data-driven machine learning model was developed based on the relationship between the experimentally measured adsorption capacities and the porous structure characteristics of the adsorbents.

## 2. Results and Discussion

The adsorption of ciprofloxacin is a complex process and is affected by several factors, such as the pH of the aqueous solution, the type of adsorbent, the solubility of the adsorbate, the temperature, and the concentration of the adsorbate solution [3,7]. As mentioned in the previous chapter, the main goal of this work is to observe how the porous properties of the carbon adsorbent affect the adsorption of ciprofloxacin when all the factors affecting the adsorption process are kept constant throughout the experimental measurements of each material.

### 2.1. Effect of pH for Adsorption

Ciprofloxacin is a zwitterionic ampholyte with two p*K*_a_ values of 5.9 (carboxyl group) and 8.9 (secondary amine) [39]. Therefore, it can exist as a cation, a zwitterion, or an anion, depending on the pH value of the environment. This means that the adsorption of ciprofloxacin is affected by the pH of the solution [40]. Several previous studies have shown that ciprofloxacin adsorption is greatest in the pH range of 5–7 [26,39,41], which corresponds mainly to the zwitterionic form of ciprofloxacin. The zwitterionic form of ampholytes usually has not only higher adsorption [26,39] but also permeation in artificial membrane systems [42,43] and a lower solubility [41] than the cationic or anionic form.

pH is an important factor in adsorption studies to obtain systematic and comparable data. To ensure that the pH of the drug substance solution prepared in MilliQ water remained constant during the adsorption experiments, the UV spectra of each adsorption solution were compared with the standard spectra measured for ciprofloxacin in different buffer solutions of known pH (see Figure S1 for details). A comparison of the spectrum maxima showed that the selected experimental conditions were stable, and the pH of the drug substance solutions prepared in the MilliQ water was ~6 during all adsorption experiments. It must be noted that UV spectra analysis was chosen to confirm the pH of the solution because pH measurements with a standard pH electrode in MilliQ solution are unreliable.

### 2.2. Adsorbent Material with Tunable Pore Size

Collecting the broad variety of nanoporous carbons was crucial to learn about the role of adsorbents pore size in the process of removing of ciprofloxacin from a pure aqueous solution. The material model system that allows systematically controllable pore sizes is nanoporous CDC, which is known as a material with a tuneable pore size distribution (PSD). Another feature of CDC, which is also important in this study, is a material with a low-functionalized surface [44,45]. Three factors mainly influence CDC’s structural order and porosity: (1) type of origin carbide, (2) carbide-to-carbon conversion temperature, and (3) post-activation conditions, if additionally applied to CDC. Considering all this, the list of carbon materials used in this study as adsorbents included 87 CDCs, which were based on seven precursor carbides (TiC, NbC, ZrC, Mo_2_C, Al_4_C_3_, SiC, and B_4_C) and were obtained by combining different synthesis conditions, for example, varied carbide chlorination at a temperature between 300 and 1100 °C [37,38,46,47,48]. Different stoichiometries and spacings of carbon atoms in the precursor carbide lattice resulted in a different pore size distribution in the resultant CDC [48,49]. The synthesis temperature mainly controls the PSD through the structural order of CDC: the higher the temperature, the higher the order and the larger the average pore size [46,50]. Catalytic additives in the reaction medium can also influence structural order. Thus, the 12 materials of this study were originally from silicon carbide (SiC) using d-metal chlorides as graphitization catalysts [47]. Furthermore, 20 carbon samples of the 87 CDCs have been post-activated using high-temperature steam etching, expanding the material’s pore size distribution [46,51]. For one material, a 5% TiO_2_ additive was used for the in situ etching during chlorination [52].

### 2.3. Adsorption Capacity of CDC-Based Nanoporous Carbons

A total of 87 nanoporous carbon materials were analysed and their measured adsorption capacity (*Q*) varied between 55 and 495 mg g^−1^ (detailed information can be found in Figure S2 and exact values at data repository found at http://dx.doi.org/10.15152/QDB.265; accessed on 24 October 2024). The difference between the results of the three replicates performed on each material was less than 10%, and the average difference was 6%. Moreover, 90% of the materials have an adsorption capacity greater than 200 mg g^−1^, of which almost half have an adsorption capacity greater than 400 mg g^−1^. Compared with the results found in the literature (see Chapter 1 for comparison), the results obtained in this work confirm that CDCs are attractive materials for adsorbing ciprofloxacin from aqueous solutions.

**Figure 2:**
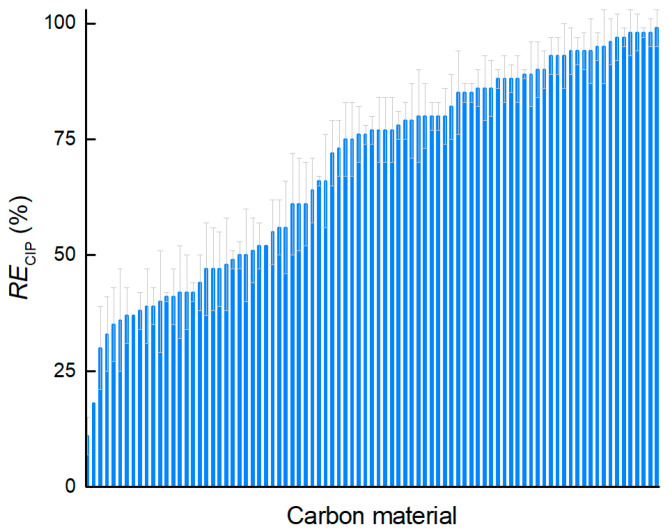
Ciprofloxacin removal efficiency (*RE*) for all 87 carbon materials.

The results show that, under the adsorption conditions used, the removal of ciprofloxacin (*RE_CIP_*) by nanoporous carbons varies significantly. The maximum efficiency for the removal of ciprofloxacin from the solution reached 99%, whereas most carbon materials were able to adsorb more than 50% ciprofloxacin (Figure 2). Only two materials with the smallest surface area were able to remove less than 20% of the drug substance, while more than a quarter of the materials removed at least 90% of the drug substance. Based on the measured adsorption capacity and removal efficiency, it can be concluded that nanoporous carbon with specific textural properties can be tuned to efficiently and effectively remove ciprofloxacin from the aqueous environment.

### 2.4. Influence of Different Texture Properties on Adsorption Capacity

**Figure 3:**
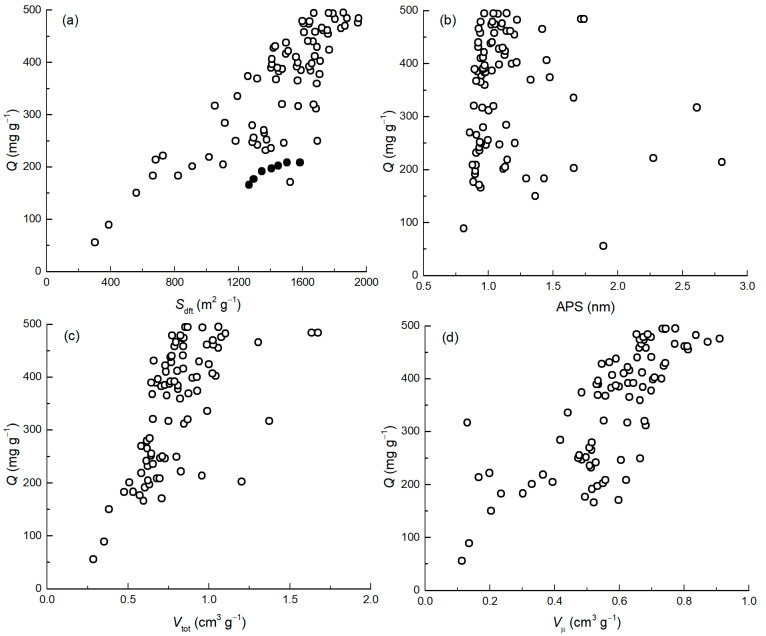
Adsorption capacity (*Q*) of carbon material vs. *S*_dft_ (**a**), APS (**b**), *V*_tot_ (**c**), and *V*_µ_ (**d**).

The relationship between specific surface area (*S*_dft_) and adsorption capacity (*Q*) shows that the higher the *S*_dft_, the higher the *Q* (Figure 3a). However, it is also seen that the materials with higher specific surface area have different adsorption capacities even when *S*_dft_ is similar.

It can be concluded that besides the specific surface area, other structure-related parameters also affect the adsorption capacity. Post-treated carbon materials, which often have high specific surface areas [46], also have high adsorption capacities, ranging from 360 to 495 mg g^−1^. The reason for the high adsorption capacity of these materials could be the slightly larger specific surface area or the wider pore size distribution. Higher adsorption capacity may also be caused by the chemically modified surface. It is known that during post-activation, the total surface oxygen content of CDC slightly increases [46].

Figure 3a shows that the six carbon materials are located below the main trend and form a semi-arc shaped group (filled marks in Figure 3a). These carbon materials have a very narrow pore size distribution and an average pore size (APS) of less than 0.9 nm, which, in turn, confirms that the pore size of the absorbent also influences adsorption. Considering the dimensions of ciprofloxacin (1.00 × 1.50 nm calculated by MarvinSketch, ver. 23.5, Chemaxon Ltd., Budapest, Hungary), we can assume that materials with very small pores cannot absorb the ciprofloxacin effectively. Figure 3b shows that most adsorbents have an APS around 1 nm, nine materials have an APS below 0.9 nm, and eight materials have an APS greater than 1.5 nm. One can observe the connection between the adsorption capacity of the micro- and the total pore volumes (Figure 3c,d)—as the pore volume increases, the adsorption capacity also increases. As a trend was observed that the higher the total pore volume of the material, the higher the adsorption capacity, while the total porosity alone does not describe the adsorption capacity. Deviating data points indicate that pore structure is also likely to be important, e.g., carbon materials have large pores that increase the overall porosity but adsorb the drug inefficiently.

### 2.5. QnSPR for Adsorption Capacity

**Figure 4:**
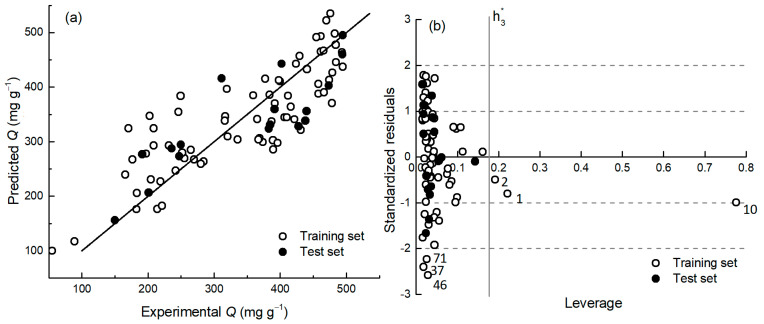
Experimental vs. predicted *Q* (**a**) and model diagnostic plot (**b**) corresponding to Equation (1).

The final three-parameter QnSPR (R^2^ = 0.70; *R*_val_^2^ = 0.70; Equation (1); Table 1; Figure 4) contains texture descriptors with a positive effect, i.e., as the value of the parameters increases, the adsorption capacity also increases. The model validation data set statistic (*R*_val_^2^ = 0.70) shows good agreement with the model training statistics (R^2^ = 0.70), indicating the reliable predictive ability of the model.

(1) $$Q=29.5+0.156\;S_{dft}+3259\;V_{N_{2}\left[1.1–1.2\right]}+865.3\;V_{N_{2}\left[3.3–3.4\right]}$$

**Figure 5:**
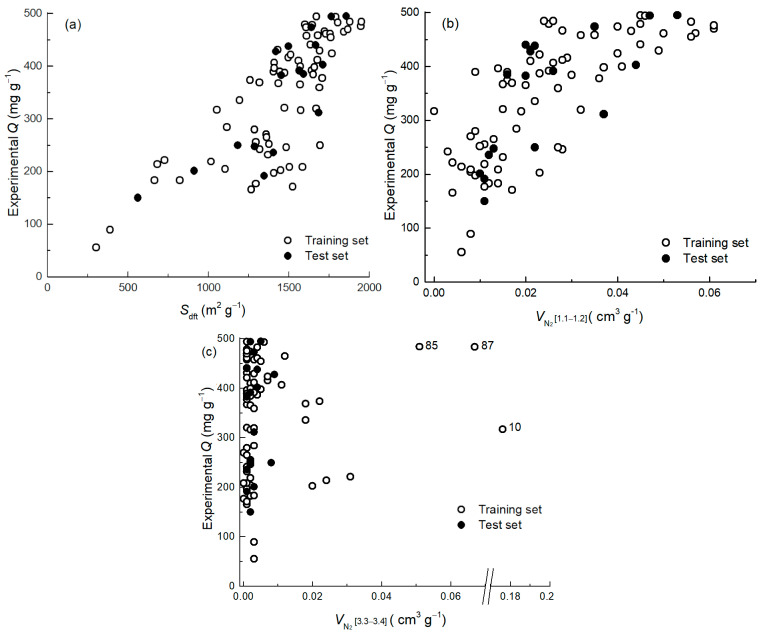
Relationship between adsorption capacity (*Q*) and individual texture descriptors: *S*_dft_ (**a**), $V_{N_{2}\left[1.1–1.2\right]}$ (**b**), and $V_{N_{2}\left[3.3–3.4\right]}$ (**c**).

The model confirms the following hypothesis presented in the previous chapter: there is a clear relationship between the adsorption capacities of the specific surface of the carbon material (Figure 5a). The second texture descriptor in the model describes a pore volume in the range of 1.1–1.2 nm (Figure 5b, $V_{N_{2}\left[1.1–1.2\right]}$]), which is in good agreement with the smallest diameter of ciprofloxacin, 1.00 nm, and has a very good positive correlation. To compensate for materials with a very low pore volume in the range of 1.1–1.2 nm, a third texture descriptor with a pore volume in the range of 3.3–3.4 nm (Figure 5c, $V_{N_{2}\left[3.3–3.4\right]}$) has been selected for the model. The addition of the descriptor is logical because, given the dimensions of ciprofloxacin, such pores could fit the drug molecules as dimers or trimers, depending on whether the largest diameter, 1.5 nm, or the smallest diameter, 1.00 nm, is considered.

### 2.6. Applicability Domain of QnSPR

The application domain of derived QnSPR for the determination of the adsorption capacity has been defined with nanoporous carbon materials and specifically with CDCs. However, because there is a wide range of structural and textural variations among the CDCs used to derive the relationship, the model is likely applicable to other similar porous materials as well.

**Figure 6:**
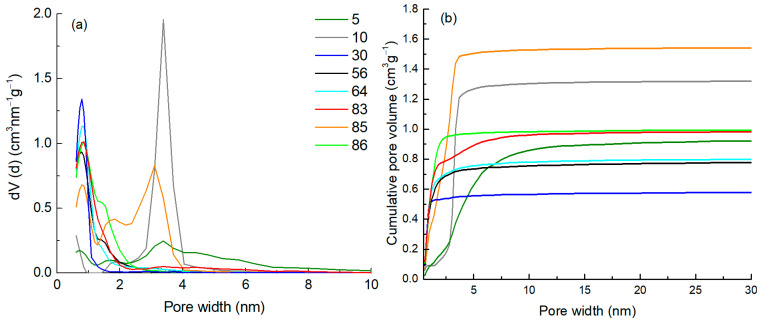
Differential (**a**) and cumulative (**b**) pore size distribution diagrams of selected carbon materials according to N_2_ adsorption.

A detailed view of the applicability domain of the model and the corresponding diagnostics are achieved with the influence plot analysis (Figure 4b), where the vertical axis shows the standardized residuals, and the horizontal axis shows the leverage (so called hat values). One can see that the model is stable, without strong outliers (no materials out of 3σ lines). The analysis of residuals (on the vertical axis) shows that three moderate outliers and three materials have higher hat-values (leverage) on the horizontal axis. Compared with other materials, one material (**10**) has a higher leverage and a relatively high Cook’s distance (Figure S3). However, it does not exceed the critical level (critical level > 1 [53]). This deserves detailed attention, revealing that the reason is found in the texture descriptors$\;V_{N_{2}\left[1.1–1.2\right]}$, which indicates that this carbon material does not have pores of this size (Figure 5b). The *V*_tot_ of this carbon material is 1.37 cm^3^ g^−1^, and the APS of this material is 2.61 nm, which is more than twice the average pore size of the materials in the set, indicating that it is a mesoporous material (see intense peak in the mesoporous region in Figure 6). The data point is located quite near the zero line of the standardized residuals (Figure 4b), suggesting that the model is also able to estimate the adsorption capacity of this type of materials with sufficient accuracy with the two other texture descriptors (*Q*_exp_ = 317 vs. *Q*_pred_ = 346 mg g^−1^). Mesoporous materials mostly have a large specific surface, giving them an excellent adsorption capacity. For example, the adsorption capacity of the two mesoporous materials (**85** and **87**; Figure 5c) is ~480 mg g^−1^, and the constructed model predicts their adsorption capacity sufficiently accurately (446 and 478 mg g^−1^, respectively). Some other materials in the data set have a certain amount of mesopores (Figure 5c) and micropores. According to the leverage, they reside well in the applicability domain of the model. Two more compounds (**1** and **2**) have higher than critical leverage but are very close to the critical leverage limit (Figure 4b). These are materials with a small specific surface area.

The three nanoporous carbon materials (**71**, **37**, and **46**) of the training data set fall within the standardized residuals range of −2σ and −3σ of the model, i.e., are moderate outliers. The analysis of the texture properties of these materials does not bring up clearly distinguishable features that would allow us to explain their deviation below the −2σ line.

## 3. Materials and Methods

### 3.1. Adsorbents

In this research, the nanoporous carbon materials of carbide origin, the so-called carbide-derived carbons (CDCs), were used as adsorbents. CDC is usually obtained by removing metal atoms from various metal or metalloid carbides by high-temperature chlorination (Equation (2)) [44,45,50].

MC_x_ + y/2Cl_2_ → MCl_y_ + xC; (M = metal or metalloid) (2)

A total of 87 CDCs of this research originated from seven different carbides (TiC (39 CDCs), NbC (3 CDCs), ZrC (3 CDCs), Mo_2_C (4 CDCs), Al_4_C_3_ (4 CDCs), SiC (30 CDCs), and B_4_C (3 CDCs)) and were synthesised at chlorination temperatures in the range of 300–1100 °C. The detailed synthesis conditions of these CDC materials are described in our earlier publications [46,47,48,49,51,52].

Most carbon materials used in this study have an ash content of less than 1%. Less than 10% of materials have an ash content of slightly more than 1% but not exceeding 2%. The average particle size of the CDC materials ranges from approximately 0.5–20 µm, which is derived from the particle size of the starting carbides as indicated in the manufacturer’s specifications.

### 3.2. Measurements of Adsorption

A solution of ciprofloxacin (C_17_H_28_FN_3_O_3_ HCl · H_2_O, 385.8 g mol^−1^; Tokyo Chemical Industry Co., Ltd., Tokyo, Japan; >98.0%) was prepared at a concentration of 100 mg L^−1^. All drug substance solutions were prepared with Milli-Q water (MQ Synergy UV water purification system, Millipore, Darmstadt, Germany) and stored in foil-lined containers protected from light. The pH of the solutions was checked with pH paper (pH-Fix 4.5–10.0 Macherey-Nagel, Dueren, Germany), and measurements of the UV spectra of the ciprofloxacin solution at different pH values were also performed (see additional information in Figure S1).

To measure the adsorption, 1.00 ± 0.04 mg of carbon material was weighed into the 5 mL test tube. Before weighing, the carbon material was vacuumed at 120 °C for 24 h. Next, 5 mL of the ciprofloxacin solution was added to the test tube. The tube was then kept on a vortex mixer (Vortex V-1 plus, BioSan, Riga, Latvia) for about 10 s to obtain a uniform suspension. After that, the tube was shaken in an orbital shaker at 300 rpm for 60 min (PSU-10i, BioSan) at 25 °C. To determine the concentration of the remaining ciprofloxacin, 1 mL of the solution was taken from the test tube with a 1 mL syringe. The solution was filtered using a PTFE syringe filter (13 mm, 0.2 μm, VWR International, Radnor, PA 19087) directly into a semi-micro-UV cuvette (BRAND, Wertheim, Germany). UV spectra measurements were performed with a SpectroStarNano UV/Vis microplate reader, which also allows for cuvette measurements (version 2.10, BMG LABTECH, Ortenberg, Germany). Full spectra were measured in the wavelength range of 220–1000 nm and with a resolution of 1 nm. For the quantification, absorbance at 355 nm was used for calculations. Before starting the measurement series, the UV/Vis spectra of the calibration solutions were measured directly in the UV cuvette, and a calibration graph was prepared. Seven consecutive 1:1 dilutions were made to construct the calibration graph (in the range of 100–0.8 mg L^−1^). Three replicate experiments were performed with each carbon material, and the difference between the obtained results was less than 10%. The obtained spectral results were analysed with MARS data analysis software (version 2.40, BMG LABTECH). All calculations and graphs were performed in Microsoft Excel (Version 2403, Build 16.0.17425.20176).

The adsorption of the ciprofloxacin by the carbon material was evaluated by the adsorption capacity (*Q*, mg g^−1^, Equation (3)) and the removal efficiency from the aqueous solution (*RE*, % Equation (4)) as follows:

(3) $$Q=\frac{\left(C_{0}-C_{e}\right)}{W}\cdot V$$

(4) $$RE=\frac{\left(C_{0}-C_{e}\right)}{C_{0}}\cdot 100\%$$

where *C*_0_ (mg L^−1^) is the initial concentration of ciprofloxacin, *C*_e_ (mg L^−1^) is the equilibrium concentration of ciprofloxacin, *W* (g) is the amount of carbon material, and *V* (L) is the volume of the ciprofloxacin solution.

The dependence of equilibrium adsorption on time was investigated on three carbon materials with different porosity. This study revealed that in the case of carbon materials with a larger specific surface area (Figure S4: **56** and **85**), the equilibrium state is reached practically immediately, whereas, in the case of a material with a smaller specific surface area (Figure S4: **30**), more time is needed. Based on these results, 60 min was chosen as the time frame for the adsorption capacity measurements of all materials in the series.

### 3.3. Texture Characteristics of Nanoporous Carbon Materials

The texture descriptors of carbon materials (Table 2) with various PSDs (see selected examples in Figure 6a) were used as independent variables. These are the volumes of N_2_ adsorption-derived pore size fractions ($V_{N_{2}[x–y]}$), specific surface area (*S*_dft_), total pore volume (*V*_tot_), volume of micropores (*V*_µ_), and average pore size (APS). All volumes of pore size fractions were calculated from the cumulative PSD data (Figure 6b) using 0.1 nm steps in the range of 0.8–5 nm pore size. The final data matrix included 47 texture descriptors. The pore size distribution and specific surface areas were calculated from N_2_ isotherms using a quenched solid density functional theory (QSDFT) equilibria model for slit-type pores [54,55,56]. All the calculations were carried out by using TouchWin 1.11 software.

### 3.4. QnSPR—Quantitive Nano-Structure–Property Relationship

The data matrix for model derivation consisted of 87 carbon materials and 49 descriptors (47 texture descriptors and 2 additional descriptors, ash content and average particle size (see Section 3.1)). When building a training and validation data set for not very large data, it is customary to start from the experimental property to be modelled. Since there is a physical relationship between the adsorption capacity and the accessible surface of the material, we decided to rank the carbon materials in the order of increasing S_dft_ and split them 80:20 into a training data set (80% of the materials) and a validation data set (20% of the materials). For this purpose, the ranked materials were divided one after the other into groups of five, and every third material of the group belonged to the validation data series and the rest to the training data series.

Before the model development, two data pre-treatment assumptions were made: (i) structural descriptors with an absolute Pearson’s correlation coefficient greater than 0.9 were excluded from the texture descriptors and (ii) the specific surface area S_dft_ was always among the descriptors because physically the (accessible) surface must be directly related to the adsorption capacity, which indeed is partially evidenced by the correlation between the specific surface area and adsorption capacity of porous carbon materials (see Figure 3a). These modifications reduced the pool of texture descriptors from 49 to 13.

QnSPR model describes the relationship between the structural characteristics of the nanomaterial and the investigated property [57]. The multilinear regression was derived using the software R (version 4.3.3) [58] package olsrr (version 0.6.0) in the graphical interface of RStudio [59]. The stepwise forward regression algorithm was used to select an optimal set of descriptors from 13 texture descriptors. The Akaike Information Criterion (AIC) [60] was used to decide the number of descriptors in the relationship. AIC finds the balance between the accuracy (given by the likelihood) and the complexity (the number of parameters) of the model.

The applicability domain of the model was discussed, and the outliers, high leverage points, and influential points were analysed [61].

The final QnSPR model (together with data) was uploaded to the QsarDB repository [62].

## 4. Conclusions

This study analysed how the porous texture of nanoporous carbon adsorbents affect their adsorption capacity. For this, the adsorption capacity for 87 carbide-derived carbon materials was measured and analysed.

Based on the adsorption measurements of the studied carbon materials, the highest adsorption capacity reached was 495 mg g^−1^, and the lowest was 55 mg g^−1^. Half of the used materials removed more than 75% and the top five more than 98% of the ciprofloxacin from an aqueous solution with a concentration of 100 mg L^−1^.

The derived three-parameter (R^2^ = 0.70) quantitative nanostructure–property relationship (QnSPR) shows a clear relationship between the texture of the nanoporous carbon material and the adsorption capacity. The analysis of the descriptors in the relationship confirms the assumption that, in addition to the specific surface area of the material, the adsorption capacity is significantly affected by the pore size distribution of the adsorbent. In addition, the texture descriptors describing the pore size distribution selected in the model show a clear relationship between the dimensions (molecular size) of the studied drug substance (ciprofloxacin) and the amount of pores with approximately the same size.

## Figures and Tables

**Table 1:** Summary of the MLR model (Equation (1)) for adsorption capacity.

Descriptor	Estimate of the Coefficient	Std. Error	t Value	*p*-Value
Intercept	2.95 × 10^1^	3.67 × 10^1^	0.80	0.42
S_dft_	1.56 × 10^−1^	3.11 × 10^−2^	5.02	4.16 × 10^−6^
$V_{N_{2}\left[1.1–1.2\right]}$	3.26 × 10^3^	6.83 × 10^2^	4.77	1.06 × 10^−5^
$V_{N_{2}\left[3.3–3.4\right]}$	8.65 × 10^2^	3.33 × 10^2^	2.60	0.01

**Table 2:** Texture descriptors used to derive the model.

Descriptor	Descriptor Explanation	Range of Value
*S*_dft_	Specific surface area	303–1951 m^2^ g^−1^
*V*_tot_	Volume of total pore	0.29–1.67 cm^3^ g^−1^
*V*_µ_	Volume of micropore	0.11–0.91 cm^3^ g^−1^
$V_{N_{2}[x–y]}$	Volume of specified pore size fraction	x–y cm^3^ g^−1^
*APS* *	Average pore size	0.81–2.80 nm

* APS = 2 × *V*_tot_/*S*_dft_.

## Data Availability

The data and model presented in this study are available at the QsarDB Repository at http://dx.doi.org/10.15152/QDB.265, accessed on 24 October 2024.

## Supplementary Materials

The following supporting information can be downloaded at: https://www.mdpi.com/article/10.3390/ijms252111696/s1.
