# Supplementary material for: Nanomaterial Texture-Based Machine Learning of Ciprofloxacin Adsorption on Nanoporous Carbon
Source: Int J Mol Sci. 2024 Oct 30;25(21):11696. doi: 10.3390/ijms252111696 (PMC11546269; doi:10.3390/ijms252111696)

Supplementary Material for

Nanomaterial Texture-Based Machine Learning of Ciprofloxacin Adsorption on Nanoporous Carbon

Maike Käärik [1,*], Nadežda Krjukova [1], Uko Maran [1], Mare Oja [1,†], Geven Piir [1] and Jaan Leis [1,2]

[1] Institute of Chemistry, University of Tartu, Ravila 14a, 50411 Tartu, Estonia.
[2] Skeleton Technologies, Sepise 7, 11415 Tallinn, Estonia
* Correspondence: maike.kaarik@ut.ee Tel.: +372-737-5279
† Current address: Department of Pharmaceutical and Pharmacological Sciences, KU Leuven, 3000 Leuven, Belgium.

Supporting Figures:

Figure S1. Spectra of ciprofloxacin test solution and ciprofloxacin solution at different pH values. The spectra have been obtained at a solution concentration of 12.5 mg L^{-1}.

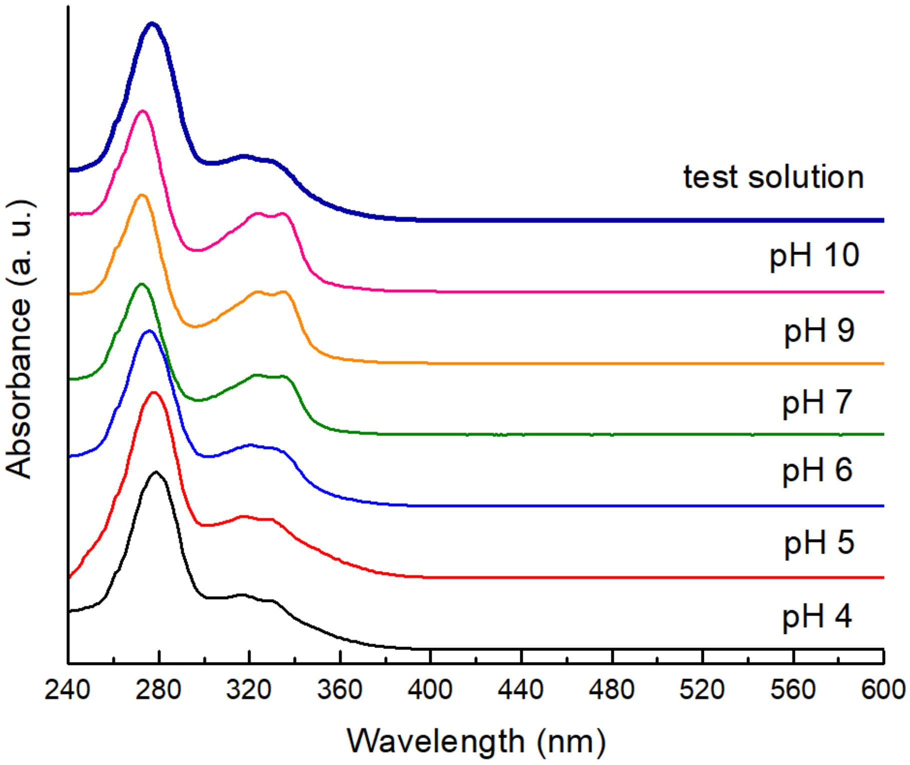

The following pH buffers were used to prepare the ciprofloxacin solutions (100 mg L^{-1}): pH 4.00 (Fluka); pH 5.00 (Sigma-Aldrich); pH 6.00 (Sigma-Aldrich); pH 7.01 (Hanna); pH 9.00 (Fluka); pH 10.01 (Hanna).

The pH of the standard buffers and ciprofloxacin solutions was measured with a pH meter (Mettler-Toledo SevenCompact S210) connected to a pH electrode (VWR). The pH meter was calibrated with the standard buffer solutions (Supelco) with pH values of 2.00; 4.00; 7.01 and 9.00. UV spectra measurements were performed with a SpectroStarNano UV/Vis microplate reader, which also allows cuvette measurements (version 2.10, BMG LABTECH). Full spectra were measured in the wavelength range 220-1000 nm and with a resolution of 1 nm. The obtained spectral results were analyzed with MARS data analysis software (version 2.40, BMG LABTECH).

Figure S2. All analyzed carbon materials with calculated standard deviation.

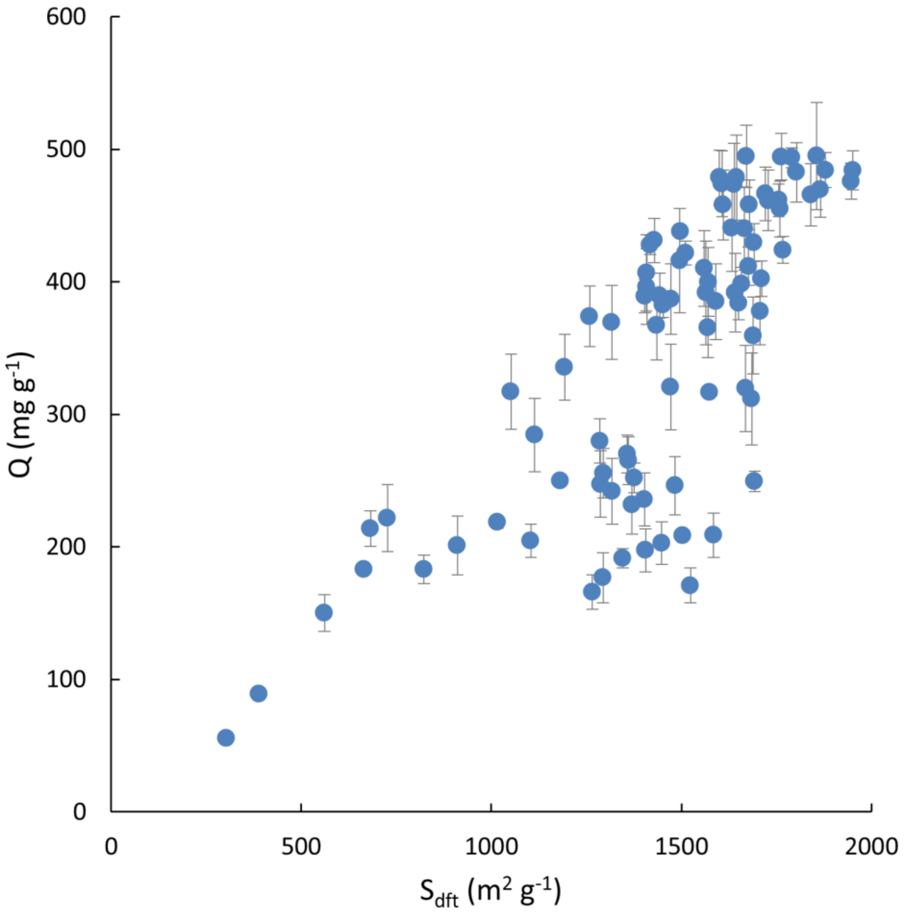

Figure S3. Cook's distance for training set 70 carbon material.

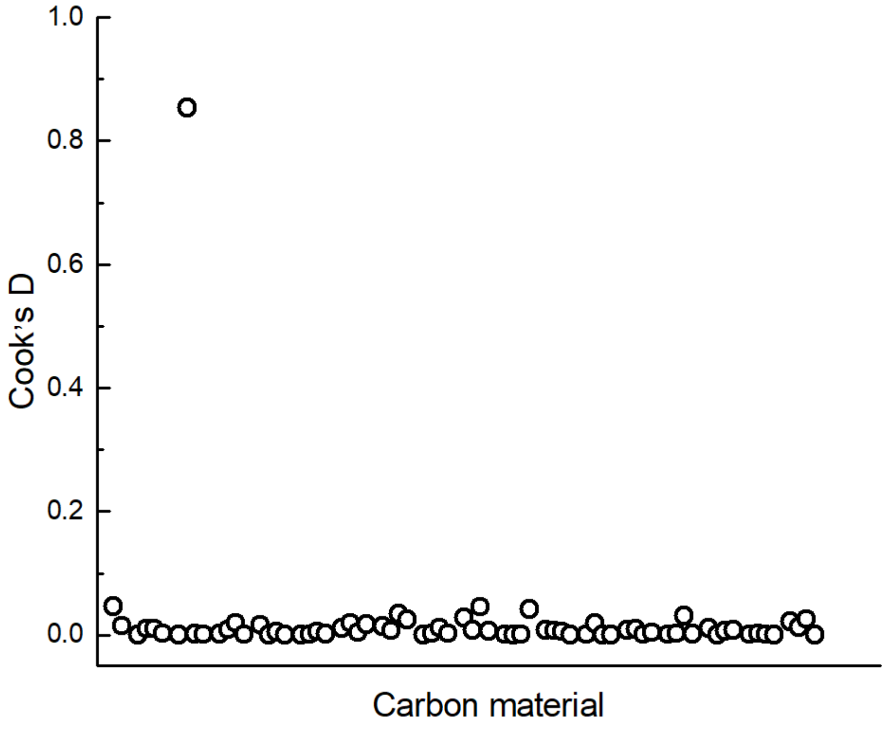

Figure S4. Adsorption capacity of ciprofloxacin at different time points.

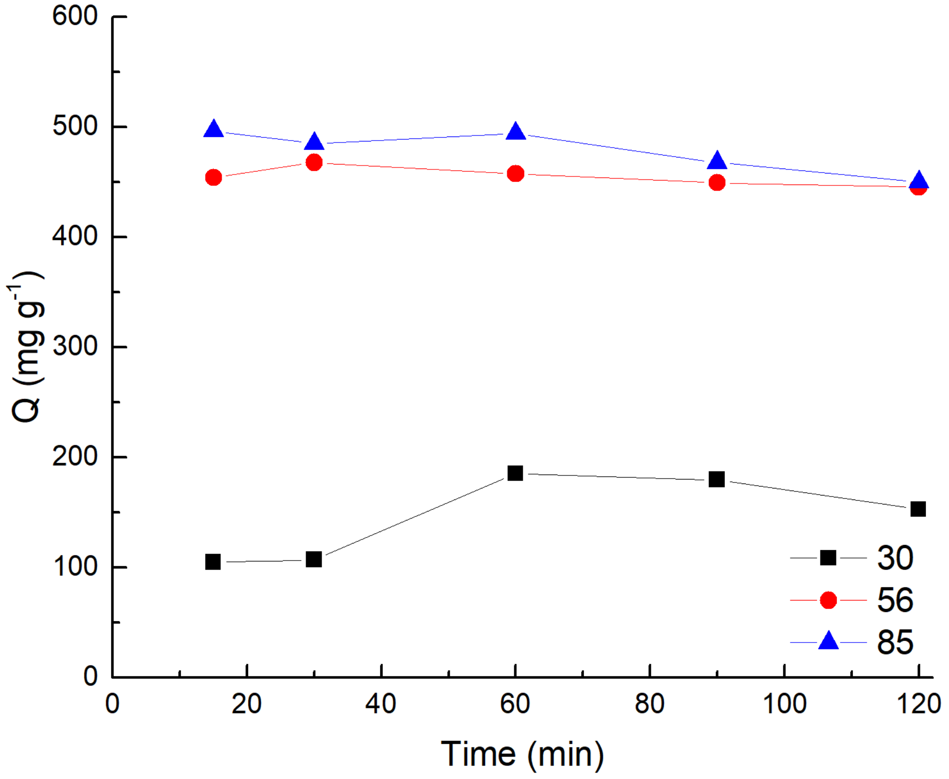

Supplement: Supplementary file 1 [file ijms-25-11696-s001.zip › ijms-3269434-supplementary.pdf]
